# The 3+3 versus model-based approach: implementation in the CAPCISUM pancreatic cancer trial

**DOI:** 10.1186/1745-6215-16-S2-P226

**Published:** 2015-11-16

**Authors:** Wendi Qian, Adrian Mander, Andrea Machin, Katy Dalchau, Pippa Corrie

**Affiliations:** 1 Cambridge Clinical Trials Unit - Cancer Theme, Cambridge, UK; 2 MRC Biostatistics Unit Hub for Trials Methodology Research, MRC Biostatistics Unit, Cambridge, UK; 3 Cambridge Cancer Centre, Cambridge University Hospitals NHS Foundation Trust, Cambridge, UK

## 

The 3+3 design is the method most commonly used to identify the maximum tolerated dose (MTD) and recommended dose for phase II testing of anti-cancer drugs. While this rule-based approach is easy to implement in practice, the true probability of toxicity at the MTD is unknown as no underlying statistical model is assumed. In the rule-based approach, decisions are made solely on information from the current dose level and may unnecessarily expose patients to sub-therapeutic doses. There has been an increased use in model-based approaches, improving study designs in practice. It is widely acknowledged by statisticians that this approach should be employed in all dose finding studies.

S-1 (Teysuno) is approved in Japan for adjuvant and palliative treatment of gastrointestinal malignancies. Clinical trials suggest a population effect exists due to metabolic differences between Asian and Caucasian populations. The CAPCISUM trial is designed to identify the optimal dose of S-1 in a Western population and to assess efficacy of S-1 compared with standard treatment in advanced pancreatic patients. The study is designed with 4 dose levels, 2 patients per level with future doses chosen using the accruing data. The operating characteristics with various prior information and models (power or logistic model) are assessed via simulations.

In practice, much work is required in the planning stage to apply the model-based approach to dose finding. Determining the level of process detail to include in the protocol, to achieve a balance between pre-specification and uncertainty of the dynamic process is a challenge.

